# Latent Network Construction for Univariate Time Series Based on Variational Auto-Encode

**DOI:** 10.3390/e23081071

**Published:** 2021-08-18

**Authors:** Jiancheng Sun, Zhinan Wu, Si Chen, Huimin Niu, Zongqing Tu

**Affiliations:** 1 School of Software and Internet of Things Engineering, Jiangxi University of Finance and Economics, Nanchang 330013, China; 2201921459@stu.jxufe.edu.cn (S.C.); 2201921462@stu.jxufe.edu.cn (H.N.); 2202020658@stu.jxufe.edu.cn (Z.T.); 2 School of Information Management, Jiangxi University of Finance and Economics, Nanchang 330013, China; 2201910057@stu.jxufe.edu.cn; 3 School of Mathematics and Computer Science, Yichun University, Yichun 336000, China

**Keywords:** time series, complex network, statistical manifold, latent space

## Abstract

Time series analysis has been an important branch of information processing, and the conversion of time series into complex networks provides a new means to understand and analyze time series. In this work, using Variational Auto-Encode (VAE), we explored the construction of latent networks for univariate time series. We first trained the VAE to obtain the space of latent probability distributions of the time series and then decomposed the multivariate Gaussian distribution into multiple univariate Gaussian distributions. By measuring the distance between univariate Gaussian distributions on a statistical manifold, the latent network construction was finally achieved. The experimental results show that the latent network can effectively retain the original information of the time series and provide a new data structure for the downstream tasks.

## 1. Introduction

The analysis of observed time series has always been a fundamental problem, both in the natural and social sciences. Depending on the application area and the problem to be solved, there are various methods for time series analysis, such as time-frequency analysis [1], classification [2], recursive graphs [3], forecasting of road traffic accidents [4], and chaotic time series analysis [5]. In recent years, as the volume of data and the complexity of systems have increased, new methods have been proposed to cope with these situations. For instance, deep learning has been successfully applied to classification [6] and forecasting of time series due to its excellent ability to automatically extract features [7,8].

In the last decade, transforming time series into complex networks and implementing time series analysis in the context of complex systems has become a critical approach. The transformation of time series into complex networks represents time series characteristics, i.e., it provides a new form of data for time series analysis. Classical approaches focus mainly on the evolutionary properties of time series. As the complexity of the system increases, it is not enough to address the evolutionary properties alone. In fact, the interaction between different components of a system or time series usually leads to an increase in complexity [9]. Therefore, we need to understand such interactions, which is the primary motivation for transforming time series into complex networks. Moreover, the transformation makes it possible to analyze time series using methods from the field of complex systems, which also provides new ways to characterize time series.

Converting a time series into a complex network usually involves three steps: extracting the components of the time series to form the nodes of the network, defining the metric of the edges, and forming the network according to certain criteria. For the extraction of network nodes, there are different approaches for different types of time series. In the case of univariate time series, the time series is usually intercepted into subsegments, which correspond to network nodes [10,11]. Lacasa et al. directly used each point in the time series as a node, forming the so-called visibility graph [12]. In transition networks, Kulp et al. proposed to map the time series to a latent Markov chain, where each state acts as a node [13]. In the case of multivariate time series, it is common practice to treat each variable as a node [14]. Based on information geometry and differential geometry theory [15,16], complex networks of time series can be constructed on Riemannian manifolds [17]. Once the nodes are determined, the next thing to be addressed is the relationship between the nodes, that is, how to determine the edges between nodes. Generally, the appropriate edge metric is chosen according to the characteristics of the nodes. For example, in the case of subsegments as nodes, the correlation coefficient or Euclidean distance can be considered to determine the edges [10]; in [13], the transition probability was used as the edge metric; and in the visibility graph, the strategy of the landscape was used to determine the edges [12]. In the final step of network construction, it is usually necessary to determine a threshold of edges, i.e., edges larger than the threshold are retained, while those smaller than the threshold are discarded.

Previous studies have focused on the definition of nodes and edges manually, which is a challenging task, especially the identification of nodes. For example, when segmenting a time series, numerous parameters such as sub-segment length, sliding window length, and sliding steps need to be determined in advance, making the optimization of the model a challenging task. In addition, time series are high dimensional and are commonly contaminated with noise. Consequently, there is an urgent need to use compressed and efficient latent features to represent time series. To address these problems, based on Variational Auto-Encode (VAE) [18], we propose in this paper to first map the univariate time series into a space of latent probability distributions and then perform the construction of latent networks. The main advantage of our method is that the nodes of the latent network can be extracted automatically, and the method is also theoretically insensitive to noise.

## 2. Materials and Methods

In this section, we first introduce the model architecture and then elaborate the training of the VAE and the construction method of the latent network, respectively.

### 2.1. Problem Statement and Framework of Proposed Model

Given a labeled dataset, $D={\left\{\left(x_{k},c_{k}\right)\right\}}_{k=1}^{K}$ consists of *K* univariate time series $x_{k}$ together with their class labels $c_{k}$, where $x_{k}$ is a sequence of real numeric values and its length is $N_{k}$, that is, $x_{k}=\left(x_{1},x_{2}\ldots x_{N_{k}}\right)$. To validate the performance of the network reconstruction, univariate time series with class labels are used here. In fact, for the network reconstruction itself, the class labels are not necessary. In addition, it is not required that the time series be of equal length.

**Figure 1:**
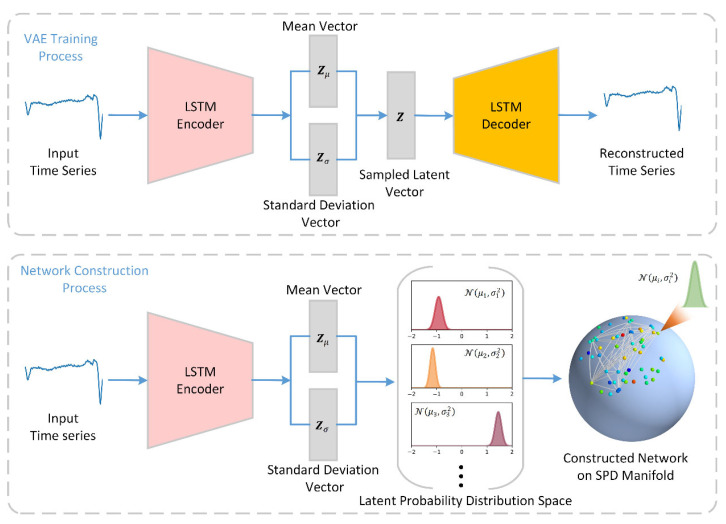
Framework of the model. The framework consists of the VAE training process (**upper part**) and the network construction process (**bottom part**).

The framework of the proposed model is shown in Figure 1, which consists of two main parts: the VAE training process and the latent network construction process. It is common practice to use Recurrent Neural Networks (RNN), Long Short-Term Memory (LSTM), and Gated Recurrent Units (GRU) to cope with time series [19]. As shown in the upper part of Figure 1, here, we chose LSTM to implement the encoding and decoding in VAE. The motivation for using VAE here was its ability to automatically extract features of the time series and generate a latent space. It is important to note that this latent space is defined by the probability distributions rather than a vector space. Therefore, VAE belongs to generative models, which are usually used to generate new data rather than using latent variables to implement downstream tasks, such as classification or regression. Some studies using latent variables to implement downstream tasks usually use either a sampled latent vector or a latent distribution as the representation of the input. When the latent distribution is used, it is also common to use only the mean vector and ignore the standard deviation vector.

Unlike previous strategies, we utilized all probabilistic information to create the latent network, i.e., including the mean and standard deviation. As shown in the bottom part of Figure 1, the input time series is first transformed into a mean vector $Z_{\mu }\in {\mathbb{R}}^{M}$ and a standard deviation vector $Z_{\sigma }\in {\mathbb{R}}^{M}$ using the trained encoder. The *i*th element in $Z_{\mu }$ and $Z_{\sigma }$ together form a univariate Gaussian probability distribution $N\left(\mu_{i},\sigma_{i}^{2}\right)$. According to the concept of information geometry [15], the probability distributions lie on a statistical manifold defined by symmetric positive definite (SPD) matrices. Finally, we derive the network by treating $N\left(\mu_{i},\sigma_{i}^{2}\right)\;\left(i=1,2,\ldots ,M\right)$ as nodes and the distance between them as edges.

### 2.2. VAE Triang Process

The structure of VAE is partially referenced in [19]. As seen in Figure 1, the VAE consists of four components: encoder, encoder-to-latent, latent-to-decoder, and decoder.

- The encoder feeds a series of input vectors $x_{k}$ into the LSTM to obtain the output vector $h_{out}^{enc}$, which is passed to the next layer;
- The role of encoder-to-latent is to map $h_{out}^{enc}$ to the latent space, i.e., a mean vector $Z_{\mu }$ and a standard deviation vector $Z_{\sigma }$, using a linear layer;
- In the latent-to-decoder phase, the sampled latent vectors are passed through a linear layer to obtain the initial state $h_{0}^{dec}$ of the decoder;
- In the final step, given the initial state $h_{0}^{dec}$, the decoder input is initialized to zero and updated using the backpropagation method; then, the output of the decoder is passed to the output layer that is a linear layer for reconstructing time series ${\hat{x}}_{k}$.

The loss function of VAE consists of two components, namely, the reconstruction error and the regularization term. The reconstruction error uses the MSE loss, while the regularization term (KL-divergence) makes the latent space more similar to the Gaussian distribution. The loss function can be expressed as:

(1) $$ℒ\left(\theta ;x_{k}\right)=-D_{KL}\left(q\left(Z|x_{k}\right)ǁp\left(Z\right)\right)+E_{q\left(Z|x_{k}\right)}\left[\log p_{\theta }\left(x_{k}|Z\right)\right]$$

where $D_{KL}$ is the KL-divergence and θ is the parameters of the model; p is the probability distribution of decoder and q is its approximation. Using the “reparametrization trick”, the resampled vector is Z=μ+σϵ with ϵ~N(0,1). Eventually, the loss function is reformulated as:

(2) $$ℒ\left(\theta ;x_{k}\right)\approx \overset{M}{\underset{m=1}{\sum }}\left(1+\log\left({\left(\sigma_{k}^{m}\right)}^{2}\right)-{\left(\mu_{k}^{m}\right)}^{2}-{\left(\sigma_{k}^{m}\right)}^{2}\right)+\frac{1}{L}\overset{L}{\underset{l=1}{\sum }}\log p\left(x_{k}|Z_{k}^{l}\right)$$

where M is the dimension of $Z_{\mu }$ and L is the number of sampled vectors Z. Here we focus on the model’s architecture, while the details of LSTM and VAE can be found in [18,20].

### 2.3. Network Construction on Manifold

Once the VAE is trained, we can use it to encode a given time series to obtain the space of the latent probability distributions. In the latent space, we use the univariate Gaussian probability distribution $N\left(\mu_{i},\sigma_{i}^{2}\right)$ as a node and then measure the distances between different distributions as edges to form the network. Some classical methods can be used directly to measure the distance between Gaussian distributions, such as Kullback–Leibler Divergence or Bhattacharyya Distance [21]. Here, we used the geodesic distance to measure the similarity between probability distributions on a statistical manifold formed by the SPD matrices. Using geodesic distances, not only can network construction be achieved, but the manifold can also provide a convenient framework and properties for downstream tasks, such as classification, regression, and dimensionality reduction [22].

According to the theory of information geometry [23], the space of D-dimensional Gaussian distributions can be embedded into the space of (D+1)×(D+1)-dimensional SPD matrices, which constitutes a manifold. For a multivariate Gaussian distribution N(μ,Σ), this embedding is defined as:

(3) $$P={\left(\det\Sigma \right)}^{-2/\left(D+1\right)}\left[\begin{array}{cc} \Sigma +\mu \mu^{T} & \mu \\ \mu^{T} & 1 \end{array}\right]$$

where P is the SPD matrix, which is localized on the manifold and corresponds to a multivariate Gaussian distribution. To measure the distance matrices $P_{i}$ and $P_{j}$, we introduce the geodesic distance on the manifold as follows [22]:

(4) $$d\left(P_{i},P_{j}\right)=\mathrm{tr}\left(\log^{2}\left(P_{i}{}^{-\frac{1}{2}}P_{j}P_{i}{}^{-\frac{1}{2}}\right)\right)$$

Considering a univariate Gaussian distribution $N\left(\mu_{i},\sigma_{i}^{2}\right)$, P is a 2×2-dimensional SPD matrix:

(5) $$P_{i}=\sigma_{i}^{-1}\left[\begin{array}{cc} \sigma_{i}+\mu_{i}^{2} & \mu_{i}\\ \mu_{i} & 1 \end{array}\right]$$

Assume that the time series $x_{k}$ is compressed by the encoder to an M-dimensional Gaussian distribution space, i.e., $Z_{\mu }\in {\mathbb{R}}^{M}$ and $Z_{\sigma }\in {\mathbb{R}}^{M}$. Using (4), we can calculate the distances between pairs of univariate distributions that form the weighted adjacency matrix $A_{k}={\left(a_{i,j}^{k}\right)}_{M\times M}$, where $a_{i,j}^{k}=d\left(P_{i},P_{j}\right)$. In some cases, to bring out the topology of the network, some edges can be removed by setting a threshold τ so that the adjacency matrix $A_{k}$ is transformed into:

(6) $${\bar{A}}_{k}=\{\begin{array}{c} a_{i,j}^{k}\;if\;a_{i,j}^{k}>\tau \\ 0\;\;\;otherwise \end{array}$$

Thus far, we have obtained the latent network $A_{k}$ of the time series $x_{k}$. That is, we have transformed the time series into a network in the latent space, which is a representation of the time series. The network can be used to study the interactions within the latent variables or as new features for downstream tasks, such as clustering and classification.

To verify the reasonableness of the network $A_{k}$, we additionally construct a network B, where $A_{k}$ is used as a node. That is, B is a network of networks. Using the class label $c_{k}$ of $x_{k}$, we can verify the reasonableness of the network $A_{k}$ from the topology of network B. To construct B, the distance between metrics $A_{k}$ is necessary. However, $A_{k}$ is not an SPD matrix, so we cannot directly apply (4) to calculate the distance. Consequently, we introduce the graph Laplacian to transform $A_{k}$ into an SPD matrix as:

(7) $$L_{k}=\left(D_{k}-A_{k}\right)+\lambda I$$

where $D_{k}=\mathrm{diag}\left(\underset{j}{\sum }a_{i,j}^{k}\right)$ is the degree matrix of $A_{k}$, λ>0 is a regularization parameter, and I is the identity matrix. Now, we have two strategies to construct the network B. One is to construct the network directly on the manifold. In this strategy, the distance between $L_{k}$ is calculated using (4) to form the adjacency matrix, i.e., $B={\left(b_{i,j}\right)}_{K\times K}$, where $b_{i,j}=d\left(L_{i},L_{j}\right)$ and K is the quantity of time series $x_{k}$. The other strategy is to project $L_{k}$ onto the tangent space of the manifold and then construct the network in the tangent space. Let M∈ℳ be the reference point defined as the mean of SPD matrices, and then the correspondence from manifold ℳ to the tangent space $T_{M}$ at M can be defined by a logarithmic mapping $\log_{M}:\;ℳ\mapsto T_{M}$ [22]:

(8) $$y_{k}=\log_{M}\left(L_{k}\right)=M^{\frac{1}{2}}\log\left(M^{-\frac{1}{2}}L_{k}M^{-\frac{1}{2}}\right)M^{\frac{1}{2}}$$

Since $T_{M}$ is a vector space, we can calculate the distance between $y_{k}$ using an appropriate metric D(,) in the Euclidean space to form the adjacency matrix, i.e., $B={\left(b_{i,j}\right)}_{K\times K}$, where $b_{i,j}=D\left(y_{i},y_{j}\right)$. In this study, we chose the second strategy to construct the network B. The reason is that $T_{M}$ is a Euclidean space, thus facilitating the application of some classical algorithms.

## 3. Experiment and Results

**Figure 2:**
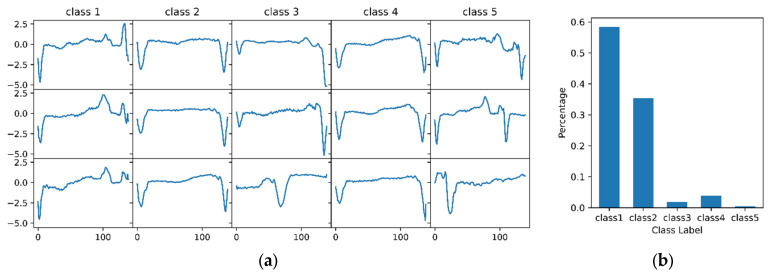
ECG5000 dataset. (**a**) Three samples in each class were randomly selected for presentation. The horizontal and vertical axes indicate the class and sample, respectively. (**b**) Percentage of sample size in each class to total sample.

In this experiment, we used the electrocardiogram (ECG) dataset named ECG5000 from the UCR archive [24], where the training and test sets have a total of 5000 samples. We first merged the training and test datasets, and then 4500 randomly selected ECG data were used to train the VAE, and another 500 were used to build the latent network. This dataset has five classes with a sample length of 140, and the class labels were used to evaluate the performance of the network construction. Some of the samples are shown in Figure 2a, where each sequence corresponds to one heartbeat. It is important to note that the samples in each class are highly imbalanced, which is demonstrated in Figure 2b. It can be seen that the first and second classes of samples dominated, with few samples from the other three categories.

**Figure 3:**
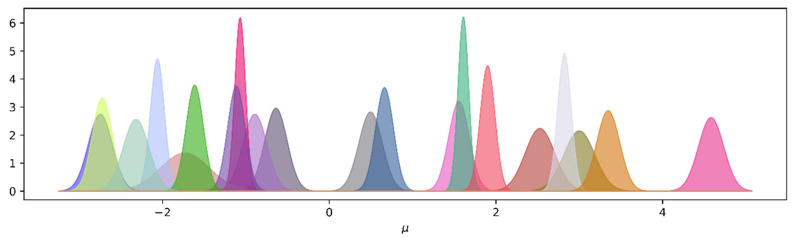
The latent probability distribution of a sequence. Different colors denote different univariate Gaussian distributions: $N\left(\mu_{i},\sigma_{i}^{2}\right)$, where $\mu_{i}$ and $\sigma_{i}^{2}$ come from the *i*th element of the latent vectors $Z_{\mu }$ and $Z_{\sigma }$, respectively.

Due to the symmetry of VAE, the LSTM in both the encoder and decoder was one layer; each layer had 96 hidden units, and the dimension of the latent vectors $Z_{\mu }$ and $Z_{\sigma }$ was 20. The learning rate was set to 0.0005, and Adam was used to update the weights. Once the VAE was trained, we then used its encoder to obtain a representation of the test data, i.e., the space of the latent probability distributions. The result of the latent space corresponding to a sequence from class 1 is shown in Figure 3.

**Figure 4:**
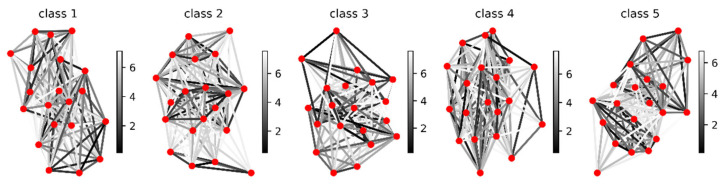
Latent networks of univariate ECG time series of different classes. Each node represents a univariate Gaussian distribution, and the edges indicate the distance between the nodes. The color bar denotes the weight of the edge.

As can be seen in Figure 3, different N have different means and standard deviations. For the time series $x_{k}$, we used (4) and (5) to calculate the distances between N, thus forming the adjacency matrix $A_{k}$. To highlight the topology of the network, we used (6) to remove most of the edges. In fact, how to determine an appropriate threshold τ in (6) has been an open problem in the construction of complex networks. Here, we retained 20% of the edges based on empirical values and visualization effects. Examples of latent networks corresponding to different time series are shown in Figure 4.

Each network in Figure 4 corresponds to a time series that belongs to a different class. By transforming time series into networks, a new data structure is provided for time series analysis. Latent networks can capture the interaction of implicit variables in time series, which is the motivation for network construction. Consequently, latent networks can provide new data structures for time series mining and analysis. Once the latent network is in place, we can use numerous techniques from complex network science to characterize the time series. For example, the topological statistics of the network are extracted to form new features to analyze time series, such as classification of sleep stages [25] and analysis of seismic sequences [26]. In addition, graph neural networks (GNNs) have recently been rapidly developed and successfully used in many fields [27]. A prerequisite to apply this powerful tool is that the input data must be graph structured. In most cases, time series do not have an explicit graph structure. Therefore, the proposed latent network provides the conditions for using GNNs to analyze time series.

**Figure 5:**
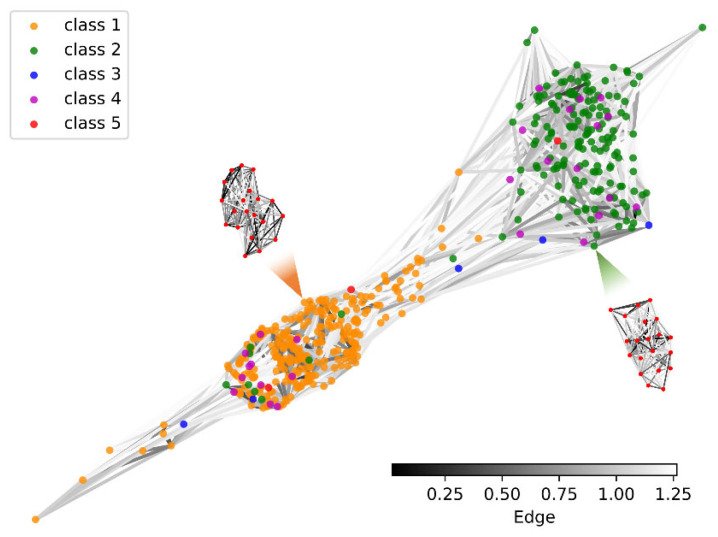
Network B of network A. Class label identifies the color of the node, and the color bar denotes the weight (Euclidean distance) of the edges.

Latent networks are representations of time series; thus, different classes of networks usually have different topologies. In Figure 4, we cannot distinguish this difference with great certainty visually due to the complexity of the network connections. To verify the plausibility of our method, using (7) and (8), we constructed a new network B based on the latent network A, and then evaluated the construction of A in conjunction with the class labels. The results for network B are shown in Figure 5.

Figure 5 can be seen as a nested network, i.e., each node in the network also represents a network. As can be seen in the figure, the nodes of class 1 and class 2 clearly form two clusters. This topology indicates that network A retains the information of the original time series, allowing samples with similar attributes to cluster together, which also validates the effectiveness of network A. However, it should also be noted that the other three classes of nodes are not effectively distinguished from each other. This phenomenon is mainly due to two factors. The first reason is the low distinguishability of these three classes of samples from the other two classes, especially the sequences of class 2 and class 4 (refer to Figure 2a). The second reason is that the percentage of samples in these three classes is too low (refer to Figure 2b), which influences the training of VAE.

## 4. Conclusions

Based on the trained VAE, our work implemented the construction of latent networks for univariate time series. We found that VAE can effectively extract the features of univariate time series and obtain intermediate representations. In addition, the constructed latent networks can effectively preserve the information of the original data and provide new data structures and analysis tools for time series analysis. The primary advantage of our work over traditional methods is that there is no need to manually identify the nodes of the network due to the ability of VAE to automatically extract the features of the time series and output the latent representation. In addition, the proposed approach is an unsupervised model, so the constructed latent network is not limited to a specific downstream task, but can provide new data structures for different tasks.

## Data Availability

The data presented in this study are available on www.cs.ucr.edu/~eamonn/time_series_data/ (accessed on 17 August 2021).
